# Body Composition Changes in Gastric Cancer Patients during Preoperative FLOT Therapy: Preliminary Results of an Italian Cohort Study

**DOI:** 10.3390/nu13030960

**Published:** 2021-03-16

**Authors:** Emanuele Rinninella, Antonia Strippoli, Marco Cintoni, Pauline Raoul, Raffaella Vivolo, Mariantonietta Di Salvatore, Enza Genco, Riccardo Manfredi, Emilio Bria, Giampaolo Tortora, Antonio Gasbarrini, Carmelo Pozzo, Maria Cristina Mele

**Affiliations:** 1 UOC di Nutrizione Clinica, Dipartimento di Scienze Mediche e Chirurgiche, Fondazione Policlinico Universitario A. Gemelli IRCCS, Largo A. Gemelli 8, 00168 Rome, Italy; emanuele.rinninella@unicatt.it; 2 Comprehensive Cancer Center, Fondazione Policlinico Universitario A. Gemelli IRCCS, Largo A. Gemelli 8, 00168 Rome, Italy; antonia.strippoli@policlinicogemelli.it (A.S.); raffaellavivolo88@gmail.com (R.V.); mariantonietta.disalvatore@policlinicogemelli.it (M.D.S.); emilio.bria@unicatt.it (E.B.); giampaolo.tortora@policlinicogemelli.it (G.T.); carmelo.pozzo@policlinicogemelli.it (C.P.); 3 Scuola di Specializzazione in Scienza dell’Alimentazione, Università di Roma Tor Vergata, Via Montpellier 1, 00133 Rome, Italy; marco.cintoni@gmail.com; 4 Dipartimento di Medicina e Chirurgia Traslazionale, Università Cattolica Del Sacro Cuore, Largo F. Vito 1, 00168 Rome, Italy; pauline.raoul1@gmail.com (P.R.); antonio.gasbarrini@unicatt.it (A.G.); 5 UOC di Radiologia Diagnostica e Interventistica Generale, Dipartimento di diagnostica per Immagini, Radioterapia Oncologica ed Ematologia, Fondazione Policlinico Universitario A. Gemelli IRCCS, Largo A. Gemelli 8, 00168 Rome, Italy; enza.genco@guest.policlinicogemelli.it (E.G.); riccardo.manfredi@policlinicogemelli.it (R.M.); 6 UOC di Medicina Interna e Gastroenterologia, Dipartimento di Scienze Mediche e Chirurgiche, Fondazione Policlinico Universitario A. Gemelli IRCCS, Largo A. Gemelli 8, 00168 Rome, Italy; 7 UOSD di Nutrizione Avanzata in Oncologia, Dipartimento di Scienze Mediche e Chirurgiche, Fondazione Policlinico Universitario A. Gemelli IRCCS, Largo A. Gemelli 8, 00168 Rome, Italy

**Keywords:** gastric cancer, muscle mass, adipose tissue, SMI, body composition, sarcopenia, neoadjuvant chemotherapy, FLOT, toxicity, personalized medicine

## Abstract

Background: The impact of the new chemotherapy, fluorouracil plus leucovorin, oxaliplatin, and docetaxel (FLOT) on body composition in gastric cancer (GC) patients remains unknown. We assessed body composition changes of GC patients receiving the FLOT regimen and their impact on treatment outcomes. Methods: Preoperative pre- and post-FLOT computed tomography (CT) scans of advanced GC patients were studied. Lumbar skeletal muscle index (SMI) and adipose indices were calculated before and after FLOT. Results: A total of 26 patients were identified between April 2019 and January 2020. Nineteen patients were sarcopenic at diagnosis. The mean BMI decreased (from 24.4 ± 3.7 to 22.6 ± 3.1; *p* < 0.0001) as well as the SMI (from 48.74 ± 9.76 to 46.52 ± 9.98; *p* = 0.009) and visceral adipose index (VAI) (from 49.04 ± 31.06 to 41.99 ± 23.91; *p* = 0.004) during preoperative FLOT therapy. BMI, SMI, and VAI variations were not associated with toxicity, Response Evaluation Criteria in Solid Tumors (RECIST), response, delay and completion of perioperative FLOT chemotherapy, and the execution of gastrectomy; a decrease of SMI ≥ 5% was associated with a higher Mandard tumor regression grade (*p* = 0.01). Conclusions: Almost three-quarters (73.1%) of GC patients were sarcopenic at diagnosis. Preoperative FLOT was associated with a further reduction in SMI, BMI, and VAI. These changes were not associated with short-term outcomes.

## 1. Introduction

Gastric cancer (GC) is the fifth most common neoplasm and the third most deadly cancer, with an estimated 783,000 deaths in 2018 in the world [1]. The epidemiology of GC varies in incidence and mortality across different geographical areas. According to the latest published data, in Italy, GC represents the sixth most frequent cause of cancer-related death with mortality rates of 18.5 for men and females 9.3 (per 100,000 inhabitants), accounting for over 9200 deaths in 2016 (5.2% of cancer deaths) [2]. GC is often diagnosed at an advanced stage and has a low 5-year survival rate. In this context, gastrectomy remains the mainstay treatment for GC. In addition to surgery, different chemotherapy strategies have been successfully developed to improve survival. The positive impact of neoadjuvant chemotherapy on survival in patients with GC has been confirmed by various meta-analyses of randomized controlled trials [3,4]. Thus, neoadjuvant chemotherapy is increasingly being applied and has also been introduced into national [5] and international guidelines [6]. Recently, the perioperative 5-fluorouracil/leucovorin/oxaliplatin/docetaxel (FLOT) regimen has been proved to be more effective in terms of clinical outcomes than previous anthracycline-based neoadjuvant chemotherapy schedules used in clinical practice [7]. Hence, in 2019, the European Society for Medical Oncology (ESMO) recommended the FLOT regimen as a perioperative treatment for patients with locally advanced (stage II–III of disease) or potentially resectable oligometastatic gastric adenocarcinoma (stage IV of disease) [8]. Al-Batran and colleagues found FLOT to be associated with significant improvement in overall survival (OS) with a median of 50 months; however, the 5-year survival rate remains less than 50%, and potentially modifiable prognostic factors are therefore warranted to personalize therapy and improve outcomes [9].

To date, the prognosis of GC patients also depends on factors other than oncologic therapy, such as functional and nutritional status. Recent studies highlighted the role of skeletal muscle mass as a prognostic marker of clinical outcomes in GC [10,11,12]. A recent meta-analysis [13] showed that low muscle mass at diagnosis is significantly associated with poorer OS, worse recurrence free-survival (RFS), and a higher risk of postoperative complications in GC patients undergoing gastrectomy. The loss of muscle mass is often due to GC itself leading to inflammatory-based mechanisms, fat-free mass breakdown, insulin-resistance, anabolic resistance, anorexia, and dysphagia resulting from the obstructive effect of the tumor mass. Furthermore, chemotherapy could play a direct role in the loss of muscle mass and adipose tissue in various cancers [14,15,16,17], especially GC [18,19,20]. Hence, in this context, the most likely hypothesis would be a deleterious effect of FLOT regimen on body composition in GC patients, even though currently there is no available data. A preliminary retrospective study was conducted to (i) assess the changes in body composition in GC patients during the preoperative part of the FLOT chemotherapy plan, and (ii) evaluate possible associations between these changes and short-term perioperative outcomes.

## 2. Materials and Methods

### 2.1. Study Design and Ethical Committee Approval

This is a retrospective study using clinical data in combination with computed tomography (CT) images. The study was approved by the Ethical Committee of Fondazione Policlinico A. Gemelli IRCCS Catholic University of the Sacred Heart (Prot. 42028/19—ID 2825). All participants signed a consent form recording their agreement to take part in the study and to have the results published anonymously.

### 2.2. Patients’ Characteristics

All patients who underwent a perioperative FLOT regimen between April 2019 and January 2020 at Fondazione Policlinico Universitario Agostino Gemelli in Rome, were identified for eligibility from a specific database. Patients were affected by gastric, lower esophageal, or gastro-oesophageal junction adenocarcinoma with stage II and III diseases (cT2 cN+ and cT3-T4 cN-/+), according to the 8th edition of the tumor, nodes, and metastases (TNM) staging system [21]. Patients were included if (1) they had a diagnosis of gastric or lower esophageal cancer, (2) they were over 18 years, (3) they had received preoperative FLOT chemotherapy, and (4) if the CT scan images had been performed in our center.

The detailed full perioperative FLOT protocol was as follows: patients received docetaxel at 50 mg/mq (intravenous injection (iv) > 2 h), oxaliplatin at 85 mg/mq (intravenous injection, iv > 2 h), leucovorin at 200 mg/mq (iv > 2 h) and fluorouracil 2600 mg/mq (iv by continuous infusion of 24 h) for 4 preoperative cycles administered every two weeks, followed by radical surgery and 4 postoperative cycles with the same treatment scheme. Surgery was scheduled for at least 4 weeks after the last dose of preoperative chemotherapy. CT scans performed at diagnosis and after the four pre-operative cycles were retrieved and reviewed. 

The medical records of the patients were also retrieved. Clinical characteristics at diagnosis including age, sex, Eastern Cooperative Oncology Group (ECOG) score, comorbidities, tumor site, tumor stage, number of cycles received, and surgical diagnostic procedure were collected. Delay of chemotherapeutic cycles, Response Evaluation Criteria in Solid Tumors (RECIST) response [22], grade 2 toxicity or greater, Mandard tumor regression grade were obtained from the same database.

### 2.3. Anthropometry, Body Composition, and Sarcopenia Assessments

Weight and height obtained from the patient’s chart were recorded by hospital staff. These anthropometric measurements were performed using a professional balance beam scale with a height rod (Seca 700 Physician’s Balance, Seca^®^). Body mass index (BMI) was calculated using the formula weight/height^2^ (in kg/m^2^). Skeletal muscle (SM), visceral fat (VAT), subcutaneous (SAT), and intramuscular adipose (IMAT) tissues were analyzed from CT images. The CT scanner, Revolution Maxima GE Healthcare (GE Healthcare^®^, United States) was used. A single DICOM image was extracted from pre- and post-neoadjuvant chemotherapy CT imaging at the level of the third lumbar vertebra (L3). The DICOM images were then exported to SliceOmatic software v5.0 (Tomovision, Montreal, Quebec, Canada). Image analysis was performed by two investigators with imaging experience and blinded to outcomes to minimize the introduction of bias. Using pre-established Hounsfield unit (HU) thresholds, areas of specific tissues were identified and quantified in cm^2^ as follows: −29 to +150 HU for SM, −190 to −30 HU for SAT, −150 to −50 HU for VAT, and −190 to −30 HU for IMAT. Skeletal muscle index (SMI), visceral adipose index (VAI), the subcutaneous (SAI) and intramuscular adipose index (IMAI) were calculated by normalizing areas of SM, VAT, SAT, and IMAT for squared height (in m^2^). According to the sex-specific consensus definitions of Fearon et al. [23], sarcopenia was defined as SMI < 55 cm^2^/m^2^ in men and <39 cm^2^/m^2^ in women. 

### 2.4. Treatment Outcomes

Treatment outcomes of interest were: (i) the delay of chemotherapeutic cycles, (ii) RECIST response, (iii) grade 2 toxicity or greater at the end of the cycles of preoperative chemotherapy, (iv) the execution of gastrectomy, (v) the completion of perioperative FLOT chemotherapy, and (vi) Mandard tumor regression grade (TRG). 

### 2.5. Statistical Analysis

Statistical analysis was performed using STATA^®^ Software (Version 14.0, Stata Corporation, College Station, TX, USA). The normal distribution of numerical data was assessed using the Kolmogorov–Smirnov test. Normally distributed numerical variables are presented as means and standard deviation (SD), non-Gaussian ones are shown as median and inter-quartile range (IQR); the categorical data are summarized as frequencies and percentages. Comparisons of proportions were performed with the Chi-square or Fisher’s exact test. Differences between normally distributed variables were compared by Student’s *t*-test, while differences between other continuous variables were assessed with Kruskal–Wallis test. A Pearson correlation was used for correlation analysis between numerical variables. Statistical significance was defined as *p* < 0.05. A post-hoc analysis of sample power was carried out to test the quality of the data obtained, due to the retrospective nature of the study.

## 3. Results

### 3.1. Patients’ Characteristics

After the exclusion of three patients without eligible CT images (performed in other centers), 26 patients were included in this study. The clinico-pathological characteristics of patients are summarized in Table 1. Of a total of 26 patients, 18 (69.2%) were male and the mean age was 63.3 ± 11.2 years. The mean BMI was 24.4 ± 3.7 kg/m^2^. Staging laparoscopy was used in 53.9% of patients. The baseline mean weight was 70.4 ± 13.3 kg. Twelve patients out of the total had comorbidities such as benign prostatic hyperplasia (*n* = 4), diabetes mellitus type 2 (*n* = 2), hypertension (*n* = 7). 

### 3.2. Body Composition Changes during Pre-Surgical FLOT Chemotherapy

**Figure 1:**
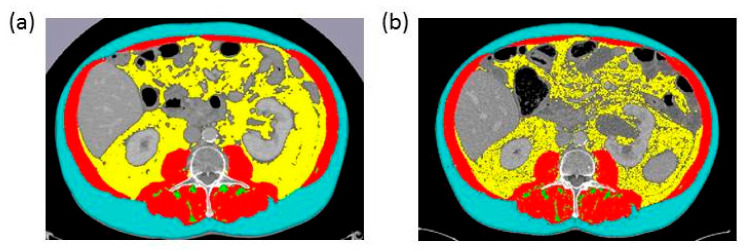
Axial CT images of the third lumbar vertebra region (**a**) before the initiation of FLOT chemotherapy and (**b**) after 4 cycles of FLOT chemotherapy in the same patient. In red, lumbar skeletal muscle area (SMA) (cm^2^); in yellow, visceral adipose tissue area (VAT) (cm^2^), in turquoise: subcutaneous adipose tissue area (SAT) (cm^2^); in green, intramuscular adipose tissue area (IMAT) (cm^2^).

Figure 1 illustrates an example of body composition measurements in CT images of one patient, which were performed before the initiation of FLOT chemotherapy and after four cycles of preoperative FLOT chemotherapy. 

Body composition data pre- and post-chemotherapy are detailed in Table 2. The mean time between the initial pre-chemotherapy CT scan and the post-chemotherapy CT scan for the entire cohort was 95.5 ± 19.6 days.

At baseline, the prevalence of sarcopenia was 73% (19/26). Between pre- and post-chemotherapy, there was a significant decrease in weight (70.4 ± 13.3 kg vs. 65.4 ± 11.6 kg, *p* = 0.0001), BMI (24.4 ± 3.7 kg/m^2^ vs. 22.6 ± 3.1 kg/m^2^, *p* < 0.0001), SMA (141.1 ± 31.9 cm^2^ vs. 134.6 ± 32.0 cm^2^, *p* = 0.01), SMI (48.74 ± 9.76 cm^2^/m^2^ vs. 46.52 ± 9.98 cm^2^/m^2^, *p* = 0.009), VAI (49.04 ± 31.06 cm^2^/m^2^ vs. 41.99 ± 23.91 cm^2^/m^2^, *p* = 0.004) and VAT (141.9 ± 92.2 cm^2^ vs. 121.3 ± 70.4 cm^2^, *p* = 0.005). The median percent change in BMI, SMI and VAI were −8.0% (range −19% to 10%), −5.0% (range −24% to 17%) and −17% (range −40% to 46%), respectively. There were no significant differences in terms of IMAT (and IMAI), SAT (and SAI) and total adipose area. 

**Figure 2:**
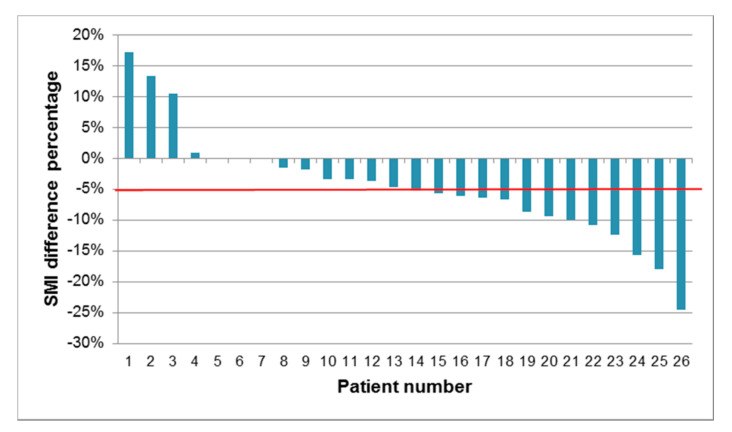
Waterfall plot demonstrating percent change in SMI between pre- and post-FLOT chemotherapy. Each bar represents one patient. The horizontal red line indicates the median % change in SMI (−5%).

Figure 2 illustrates the percent change in SMI between pre- and post-FLOT chemotherapy for each patient in a waterfall plot. 

### 3.3. Correlations of BMI with SMI and VAI

**Figure 3:**
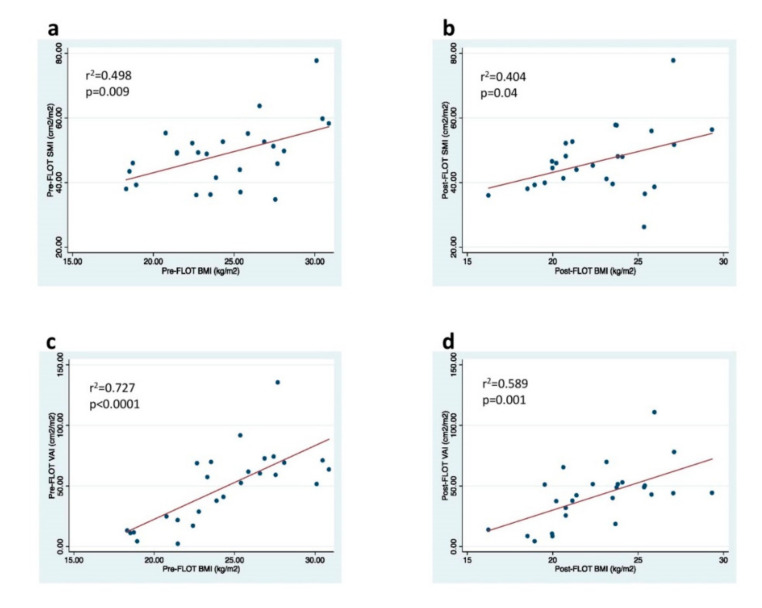
Correlation between BMI, SMI, and VAI before and after pre-surgical FLOT chemotherapy in patients with GC. Pearson correlation changes in (**a**) pre-FLOT BMI and SMI, (**b**) post-FLOT BMI and SMI, (**c**) pre-FLOT BMI and VAI, (**d**) post-FLOT BMI and VAI. Abbreviations: BMI, body mass index; FLOT, fluorouracil plus leucovorin, oxaliplatin, and docetaxel; r, Pearson’s linear correlation coefficient; SMI, skeletal mass index; VAI, visceral adipose index.

A significant positive correlation was found between BMI and SMI before and after completion of pre-surgical FLOT chemotherapy (r^2^ = 0.498, *p* = 0.009, Figure 3a; r^2^ = 0.404, *p* = 0.04; Figure 3b). BMI was also significantly correlated with VAI before and after completion of pre-surgical FLOT chemotherapy (r^2^ = 0.727, *p* < 0.0001, Figure 3c; r^2^ = 0.589, *p* < =0.001, Figure 3d). 

### 3.4. Impact of Body Composition Variations on Treatment Outcomes

Given that BMI, SMI, and VAI significantly decreased during preoperative FLOT therapy, their median values have been used as cut-off points to assess if these reductions could be associated with clinical short-term endpoints. Delay in chemotherapy cycles was found in 3 (11.5%) patients, while toxicity > grade 2 was found in 9 (34.6%) patients. Toxicities >grade 2 were mainly fatigue (*n* = 1), alopecia (*n* = 1), paraesthesia (*n* = 1), neutropenia (*n* = 1), diarrhea (*n* = 2), nausea (*n* = 1), stomatitis (*n* = 1) and neurotoxicity (*n* = 1). Stable disease (SD), according to RECIST, was found in 20 (76.9%) patients at the end of the four cycles of chemotherapy.

Overall, 23 patients out of the total underwent surgery while 20 patients of the total completed the four post-surgical cycles of the FLOT regimen. No significant associations between baseline sarcopenia status, a decrease ≥ of the median value of BMI, SMI, and VAI, and the aforementioned treatment outcomes were observed. However, Mandard TRG was significantly associated with SMI decrease (*p* = 0.01). The results are detailed in Table 3.

## 4. Discussion

These preliminary data showed that almost three-quarters of GC patients were found to be sarcopenic before initiating preoperative FLOT chemotherapy. Moreover, there were significant reductions in BMI, SMI, and VAI during FLOT treatment. However, these body composition variations did not impact early clinical outcomes such as delay of chemotherapy cycles, RECIST response, and grade 2 toxicity or greater; also, they did not impact the execution of gastrectomy and the completion of perioperative FLOT chemotherapy. Only a SMI decrease higher than 5% was significantly associated with minor Mandard TRG.

To the best of our knowledge, this is the first study assessing the variations in body composition during FLOT protocol, which is the new European standard perioperative treatment for GC patients. L3 CT-scan analyses were chosen as the method to detect body composition changes in adult GC patients before and after FLOT chemotherapy [24]. Muscle mass can also be measured with different methodologies other than CT imaging, such as dual-energy X-ray absorptiometry, bioimpedance analysis, and a combination of anthropometric measurements [24]. However, CT scan images are already available for analysis due to their need for diagnosis and follow-up visits.

Body composition is known to dramatically impact clinical outcomes in cancer patients [13,14,15,16,17]. Recent literature has reported a high prevalence of low muscle mass at diagnosis reaching 50%–60% in patients with GC [13,25], in line with the results of this study. The majority of studies (including ours) used a low SMI as a marker of sarcopenia, according to the European Working Group on Sarcopenia in Older People 2 (EWGSOP2) [24]. Reduced muscle mass is also one of the phenotypic criteria proposed by the Global Leadership Initiative on Malnutrition (GLIM) for the diagnosis of malnutrition [26]. Muscle wasting is caused by multifactorial factors reflecting nutritional deficiency, systemic inflammation, and catabolism related to both tumor and aging. At diagnosis, patients can suffer from dysphagia, nausea, vomiting, diarrhea, epigastric pain, and consequently reduce their caloric intake, exacerbating the impairment of nutritional status due to disease itself.

Preoperative FLOT chemotherapy may further reduce SMI in GC patients and our results are consistent with findings of recent previous studies using other chemotherapy treatments [18,19,20]. Indeed, Den Boer et al. [19] retrospectively assessed CT images of 199 patients with gastro-oesophageal cancer before and after neoadjuvant chemotherapy (epirubicin-cisplatin-capecitabine), which showed a significant reduction in SMI. Moreover, they found a significant reduction in SAI and a slight depletion in VAI. In a cohort study of 41 advanced gastric cancer patients treated with neoadjuvant chemotherapy (cisplatin-S-1 or docetaxel-cisplatin-S-1 or S-1-oxaliplatin), Matsuura et al. found no difference in BMI, but a significant reduction in SMI [20]. Another study of 47 patients with oesophagogastric cancer showed significant reductions in fat-free mass and fat mass calculated from CT scans before and after 3 cycles of preoperative epirubicin/cisplatin/5-fluorouracil [18]. The mechanisms by which chemotherapy, in particular, the FLOT regimen, may impact body composition remain poorly understood. A possible explanation is that the acceleration of muscle mass loss could be associated with the production of pro-inflammatory cytokines including IL-6, IL-10, and TNF-alpha [27]. A recent prospective study confirmed this hypothesis, suggesting an association of chemotherapy-induced sarcopenia with serum C-reactive protein, IL-8, and TNF-alpha levels [28]. Both chemotherapy and cancer molecular pathways could activate various mechanisms associated with movement disorders, necrosis, muscle cell death, muscle weakness, and muscle damage [29]. These metabolic and muscular derangements could potentially play a role in cancer therapy, inducing toxicity during treatment. Indeed, to date, the calculation of doses of drugs are based only on body surface area (of each patient), and not on muscle and fat mass.

Recently, adipose tissue has also emerged as a prognostic marker in cancer patients, although with substantial differences according to its anatomical distribution. SAT is considered a positive prognostic factor in cancer patients given its function of energetic storage [30]; indeed, rapid depletion of SAT has been independently associated with worse OS in patients affected by hepatocellular carcinoma during sorafenib treatment [31]. On the other hand, IMAT content is considered a marker of poor skeletal muscle quality and a prognostic factor of negative short- and long-term outcomes in cancer patients [17,32]. Finally, VAT has been shown as an independent risk factor for postoperative complications (pneumonia and intra-abdominal abscess) in GC patients undergoing gastrectomy [33]. Our study demonstrated significant reductions of VAT in GC patients following a preoperative FLOT regimen. This is in line with the results of a larger recent Chinese retrospective cohort study that enrolled 157 GC patients undergoing surgery after neoadjuvant therapy with several protocols (also including chemoradiotherapy) [34]: the authors reported a significant SAT and VAT loss during neoadjuvant treatments, both were associated with a poorer OS and disease-free survival (DFS) in the univariate analysis. The combination of a marked VAT loss with a marked SAT loss was an independent predictor of OS and DFS. This evidence may suggest the need to provide high caloric nutritional support during neoadjuvant therapy in GC patients. Our study did not report significant differences in IMAT and SAT during preoperative FLOT therapy. However, our cohort only underwent the FLOT regimen as neoadjuvant treatment. Moreover, these results are not conclusive and further studies with longer observation periods are needed to confirm these data and correlate them with clinical outcomes. 

Consistent with previous studies [18,35], this study showed a significant positive correlation between BMI and both SMI and VAI, before and after the FLOT regimen. Thus, a low BMI is often associated with preoperative sarcopenia and low VAT. Although BMI does not provide information about muscle and fat distribution, this anthropometric index is widely used in clinical practice and remains a reliable tool to measure nutritional status and to evaluate prognosis for GC patients [36]. Indeed, BMI is a criterion included in various malnutrition assessment tools such as the Nutrition Risk Screening (NRS-2002), Mini Nutritional Assessment (MNA) as well as recent Global Leadership Initiative on Malnutrition (GLIM) criteria [26].

A peculiar association between Mandard TRG and SMI was found (*p* < 0.01). The Mandard TRG is a histological score of response after neoadjuvant treatment that is commonly used in locally advanced gastrointestinal malignancies. It proposes five stages based on the number of regressive changes (such as fibrosis), stromal and cytological alterations [37]: the lower is the grade, the better is the response. In our preliminary cohort, 6 out of 7 patients with a worse response reported a SMI decrease ≥5%. Although sarcopenia is well-known to be associated with worse treatment response in terms of reduced OS and RFS in GC [13], its impact on histologic response has not yet been elucidated. It can be argued that muscle mass may be involved in the drug pharmacokinetics and the synthesis of several acute-phase proteins [38] needed for the treatment response, but these results need to be confirmed due to the small sample size. Moreover, a greater pathological response may correspond to a reduction in dysphagia, and consequently, a better nutritional intake.

On the other hand, this study reported no significant association of body composition changes (BMI, SMI, and VAI) with treatment outcomes in terms of delay of chemotherapeutic cycles, RECIST response, toxicity, and completion of perioperative FLOT treatment, confirming the findings of similar studies [18,20]. In particular, no association was found between loss of muscle mass during neoadjuvant chemotherapy and non-completion of chemotherapy and 12-month mortality in 47 GC patients [18]. However, it is known that both BMI and SMI are useful prognostic factors for long-term outcomes such as OS in GC after gastrectomy [13,35]. Indeed, a recent retrospective cohort study of 305 GC patients [35] demonstrated that preoperative low BMI and low SMI were independent prognostic factors for the long-term OS. Furthermore, the association of low SMI at diagnosis with a poorer OS was assessed in more than 5600 GC patients [13]. After surgery, a majority of patients experience a significant weight loss during the first two months due to postoperative complications, poor nutrition, prolonged hospital stay, and deterioration of quality of life [39,40]. In this context, further prospective studies should evaluate whether preoperative changes in body composition can impact postoperative complications and quality of life. 

Pre-habilitative interventions from the initiation of preoperative FLOT chemotherapy involving nutritional support could be useful to early mitigate the reduction in BMI and SMI in GC patients and possibly improve survival outcomes. However, the number of studies evaluating the impact of nutritional support on muscle and fat mass is still limited in GC [41]. In colorectal cancer, a retrospective cohort study of our team proposed a nutritional protocol within Enhanced Recovery After Surgery (ERAS) programs in colorectal surgery, starting from preadmission [42]. Length of hospital stay was significantly reduced in the ERAS + NutriCatt protocol compared with the standard ERAS program, as well as postoperative complications. Moreover, cost-effectiveness analyses indicated savings in the ERAS + NutriCatt protocol. 

This study had some limitations. First of all, it was a retrospective, single-center study with a small sample size. Indeed, this was a preliminary study on a cohort of GC patients undergoing FLOT therapy before gastrectomy. Moreover, there was no control group, i.e., GC patients that did not undergo the neoadjuvant FLOT regimen, due to obvious unethical reasons. Additionally, potential selection bias may have influenced our results due to differences between patients whose CT-scans were available and those excluded for lack of available images. In this regard, we chose to select only patients whose CT-scans were performed in our center for this study, to avoid technical biases. Finally, muscle function has not been assessed; further studies should include a handgrip strength test as a functional test.

Despite these limitations, this is the first study to evaluate body composition changes and their impact on short-term outcomes during the FLOT regimen as preoperative treatment in GC patients. Given the recent ESMO recommendation as a new standard therapy for patients with locally advanced or potentially resectable oligometastatic GC, this study provides the first evidence of body composition changes during FLOT therapy.

## 5. Conclusions

Sarcopenia was found in 73% of GC patients initiating neoadjuvant chemotherapy and that preoperative FLOT regimen was associated with further reductions in SMI and a decrease in BMI and VAI. These changes in body composition were not associated with adverse treatment outcomes in a short period of observation. Prospective studies with a large sample size, following GC patients for a longer period are needed to further evaluate the effects of perioperative FLOT chemotherapy on body composition and functional muscle performance, and whether these variations impact surgical and post-FLOT chemotherapy outcomes as well as survival outcomes. Finally, further studies are warranted to prospectively investigate the effects of personalized nutritional support on the FLOT-induced body composition changes during the full period of care.

## Figures and Tables

**Table 1:** Patients’ characteristics.

Variables.	Total of Patients (*n* = 26)
Age (years), mean ± SD	63.3 ± 11.2
Gender, *n* (%)	
Male	18 (69.2)
Female	8 (30.8)
Weight (kg), mean ± SD	70.4 ± 13.3
Height (cm), mean ± SD	169.7 ± 8.3
BMI (kg/m^2^), mean ± SD	24.4 ± 3.7
Mandard TRG, mean ± SD	3.1± 0.9
Pathological tumor stage (ypT), *n* (%)	
ypT0	
ypT1	0 (0)
ypT2	4 (15.4)
ypT3	5 (19.2)
ypT4	6 (23.1)
Missing	9 (34.6)
	2 (7.7)
Pathological nodal stage (ypN), *n* (%)	
ypN0	8 (30.8)
ypN1	5 (19.2)
ypN2	4 (15.4)
ypN3	7 (26.9)
Missing	2 (7.7)
Presence of metastases, *n* (%)	
Yes	2 (7.7)
No	24 (92.3)
HER 2, *n* (%)	
Positive	3 (11.5)
Negative	23 (88.5)
ECOG performance status, *n* (%)	
0	23 (88.5)
1	3 (11.5)
Diagnostic laparoscopy, *n* (%)	
Yes	14 (53.9)
No	12 (46.1)
Echo-endoscopy, *n* (%)	
Yes	3 (11.5)
No	23 (88.5)
Other neoplasms treatments, *n* (%)	
Yes	3 (11.5)
No	23 (88.5)
Previous abdominal surgery	
Yes	3 (11.5)
No	23 (88.5)
Presence of comorbidities, *n* (%)	
Yes	12 (46.2)
No	14 (53.8)
BPH, *n* (%)	4 (15.4)
Yes	22 (84.6)
No	
Diabetes, *n* (%)	
Yes	2 (7.7)
No	24 (92.3)
Hypertension, *n* (%)	
Yes	7 (26.9)
No	19 (73.1)

Abbreviations: BMI, body mass index; BPH, benign prostatic hyperplasia, ChT, chemotherapy; cm, centimeter; ECOG, Eastern Cooperative Oncology Group; FLOT, fluorouracil plus leucovorin, oxaliplatin, and docetaxel; HER, human epidermal growth factor receptor 2; kg, kilogram; SD, standard deviation; TNM, tumor (T), nodes (N), and metastases (M).

**Table 2:** Body composition variations between pre- and post-chemotherapy in patients (*n* = 26) following FLOT chemotherapy for gastric cancer.

Variables (mean ± SD or *n*, %)	Pre-chT	Post-chT	*p*-Value
Weight, kg	70.4 ± 13.3	65.4 ± 11.6	**0.0001**
BMI, kg/m^2^	24.4 ± 3.7	22.6 ± 3.1	**<0.0001**
SMA, cm^2^	141.1 ± 31.9	134.6 ± 32.0	**0.01**
IMAT, cm^2^	6.69 ± 4.57	6.52 ± 4.22	0.75
VAT, cm^2^	141.9 ± 92.2	121.3 ± 70.4	**0.005**
SAT, cm^2^	132.9 ± 58.9	135.1 ± 59.4	0.79
Total adipose area, cm^2^	281.5 ± 136.8	262.9 ± 108.8	0.15
SMI, cm^2^/m^2^	48.74 ± 9.76	46.52 ± 9.98	**0.009**
IMAI, cm^2^/m^2^	2.35 ± 1.57	2.28 ± 1.52	0.73
VAI, cm^2^/m^2^	49.04 ± 31.06	41.99 ± 23.91	**0.004**
SAI, cm^2^/m^2^	46.18 ± 19.88	47.29 ± 21.06	0.69

Abbreviations: BMI, body mass index; chT, chemotherapy; IMAI, intramuscular adipose index; IMAT, intramuscular adipose tissue; SAI, subcutaneous adipose index; SAT, subcutaneous adipose tissue; SMA, skeletal muscle mass area; SMI, skeletal muscle index; VAI, visceral adipose index; VAT, visceral adipose tissue. Significant variations results (*p* > 0.05) are indicated in bold letters.

**Table 3:** Body composition changes and treatment outcomes.

		Decrease in BMI (%)			Decrease in SMI (%)			Decrease in VAI (%)		
	Total (*n* = 26)	≤8 (*n* = 13)	>8 (*n* = 13)	*p*-value	≤5 (*n* = 13)	>5 (*n* = 13)	*p*-value	≤17 (*n* = 13)	>17 (*n* = 13)	*p*-value
**Delay ChT, *n* (%)** Yes No										
									
	3 (11.5)	1 (33.3)	2 (66.7)	0.54	1 (33.3)	2 (66.7)	0.54	1 (33.3)	2 (66.7)	0.54
	23 (88.5)	12 (52.2)	11 (47.8)		12 (52.2)	11 (47.8)		12 (52.2)	11 (47.8)	
**RECIST response, *n* (%)** Stable Partial										
									
	6 (23.1)	2 (33.3)	4 (66.7)	0.59	3 (50.0)	3 (50.0)	1.0	3 (50.0)	3 (50.0)	0.97
	20 (76.9)	11 (55.0)	9 (45.0)		10 (50.0)	10 (50.0)		10 (50.0)	10 (50.0)	
**Toxicity, *n* (%)** ≥grade 2 <grade 2										
									
	9 (34.6)	5 (55.6)	4 (44.4)	0.68	3 (33.3)	6 (66.7)	0.21	4 (44.4)	5 (55.6)	0.72
	17 (65.4)	8 (47.1)	9 (52.9)		10 (58.8)	7 (41.2)		9 (52.9)	8 (47.1)	
**Execution of gastrectomy, *n* (%)** Yes No										
									
									
	23 (88.5)	12 (52.2)	11 (47.8)	0.54	12 (52.2)	11 (47.8)	0.54	12 (52.2)	11 (47.8)	0.54
	3 (11.5)	1 (33.3)	2 (66.7)		1 (33.3)	2 (66.7)		1 (33.3)	2 (66.7)	
**Completion of the post- FLOT ChT, *n* (%)** Yes No										
									
									
									
	20 (76.9)	11 (55.0)	9 (45.0)	0.35	11 (55.0)	9 (45.0)	0.65	11 (55.0)	9 (45.0)	0.35
	6 (23.1)	2 (33.3)	4 (66.7)		2 (33.3)	4 (66.7)		2 (33.3)	4 (66.7)	
**Mandard TRG, *n* (%)** ≤3 (major) >3 (minor) NR										
									
	15 (57.7)	9 (60.0)	6 (40.0)	0.45	9 (60.0)	4 (40.0)	0.01	10 (66.7)	5 (33.3)	0.09
	7 (26.9)	3 (42.9)	4 (57.1)		1 (14.0)	6 (86.0)		2 (28.6)	5 (71.4)	
	4 (15.3)	-	-		-	-		-	-	

Abbreviations: ChT, chemotherapy; FLOT, fluorouracil plus leucovorin, oxaliplatin, and docetaxel; NR, not reported; RECIST, response evaluation criteria in solid tumors; SMI, skeletal muscle index; TRG, Tumor Regression Grade. Values in parentheses are percentages. Significant results (*p* > 0.05) are indicated in bold letters.

## Data Availability

The data presented in this study are available on request from the corresponding author for any academic use upon citation of this article. The data are not publicly available due to privacy and permission restricted to publication of this article only.
